# Microscopic Analysis of Nuclear Speckles in a Viviparous Reptile

**DOI:** 10.3390/ijms25105281

**Published:** 2024-05-12

**Authors:** Jeniffer Acosta-Cárdenas, Luis Felipe Jiménez-García, Sarai de Jesús Cruz-Gómez, Ana Paulina Mendoza-von der Borch, María de Lourdes Segura-Valdez

**Affiliations:** 1 Laboratorio de Nanobiología Celular, Departamento de Biología Celular, Facultad de Ciencias, Universidad Nacional Autónoma de México—UNAM, Mexico City 04510, Mexico; jeny91ac@gmail.com (J.A.-C.); luisfelipe_jimenez@ciencias.unam.mx (L.F.J.-G.); saraicruz@ciencias.unam.mx (S.d.J.C.-G.); paulina_mendoza@ciencias.unam.mx (A.P.M.-v.d.B.); 2 Posgrado en Ciencias Biológicas, Universidad Nacional Autónoma de México, Mexico City 04510, Mexico

**Keywords:** cell nucleus, nuclear speckles, reptile, splicing factors, SR proteins, ribonucleoproteins, IGC

## Abstract

Nuclear speckles are compartments enriched in splicing factors present in the nucleoplasm of eucaryote cells. Speckles have been studied in mammalian culture and tissue cells, as well as in some non-mammalian vertebrate cells and invertebrate oocytes. In mammals, their morphology is linked to the transcriptional and splicing activities of the cell through a recruitment mechanism. In rats, speckle morphology depends on the hormonal cycle. In the present work, we explore whether a similar situation is also present in non-mammalian cells during the reproductive cycle. We studied the speckled pattern in several tissues of a viviparous reptile, the lizard *Sceloporus torquatus*, during two different stages of reproduction. We used immunofluorescence staining against splicing factors in hepatocytes and oviduct epithelium cells and fluorescence and confocal microscopy, as well as ultrastructural immunolocalization and EDTA contrast in Transmission Electron Microscopy. The distribution of splicing factors in the nucleoplasm of oviductal cells and hepatocytes coincides with the nuclear-speckled pattern described in mammals. Ultrastructurally, those cell types display Interchromatin Granule Clusters and Perichromatin Fibers. In addition, the morphology of speckles varies in oviduct cells at the two stages of the reproductive cycle analyzed, paralleling the phenomenon observed in the rat. The results show that the morphology of speckles in reptile cells depends upon the reproductive stage as it occurs in mammals.

## 1. Introduction

The cell nucleus is a highly compartmentalized organelle. Nuclear compartments are visualized by light and electron microscopy. By light microscopy, the nucleus is shown to contain several nuclear bodies, including nucleoli, Cajal bodies, and speckles [1,2].

Nuclear speckles are interchromatin compartments enriched in splicing factors [3]. They were described first in mammalian cells in culture and some tissues [4,5] but currently are known as well-established compartments within the nuclei of eukaryotes [6]. They have been described in protists, plants, and invertebrate and vertebrate animals, although with different morphological and ultrastructural patterns as well as different possible functions according to the cell type and biological model in study [7,8,9,10,11,12,13]. Morphologically, speckles are surrounded by a diffuse staining environment [14,15], and their number, morphology, and size vary in relation to gene expression [16,17]. The speckles are composed of ribonucleoproteins (RNP) and non-ribonucleoproteins splicing factors; among the latter are the SR family of splicing factors [18,19,20,21]. Their function is associated with splicing activities and gene expression regulation at different levels within the nucleoplasm [1,2,22].

Ultrastructurally, the speckles constitute the Interchromatin Granule Clusters (IGCs), while the diffuse staining environment that connects them represents the Perichromatin Fibers (PFs), the sites of active transcription [2,15,18,23]. The mammal IGCs are clusters of 0.8–1.8 μm in diameter or 0.3–3.0 μm [24], formed with interconnected granules of 20–25 nm [1]. On the other hand, the PFs are fibrillar structures of 3–5 nm width, present in the periphery of the IGCs or other nucleoplasm regions [3,23,25,26], but generally, they are always associated with the periphery of compact chromatin [25,27,28,29].

In mammals, the morphology of speckles is different depending on the transcriptional and splicing activities of the cell [16,30]. The speckles become rounded and more compact when cells are treated with RNA polymerase II inhibitors such as α-amanitin or DRB [31,32,33,34,35]. When transcription is activated, speckle constituents are recruited from the speckles to the sites of active transcription [16,36].

Up to today, nuclear speckles are seen as biomolecular condensates that self-assemble through a liquid–liquid phase separation thermodynamic process [2,37,38,39,40]. The majority of current studies on nuclear speckles are devoted to elucidating their molecular structure and analyzing the chemical–physical mechanisms that support it [41,42,43], as well as to showing evidence supporting the role of speckles in the regulation of cellular expression [2,44,45,46,47]. Moreover, there is a developing field regarding the implications of speckles in several diseases and viral infection mechanisms including HIV [48,49,50,51,52,53]. As a group, we are interested in determining whether the structure and morphological dynamics of the nuclear speckles can be considered a common trait of phylogenetic-related groups. That is why, in this paper, we analyzed whether the morphology of speckles changes during the reproductive cycle of non-mammalian vertebrate cells, similar to those observed in the rat [4]. We documented here that a similar situation observed in rats is present in the oviductal cells of the viviparous lizard *Sceloporus torquatus* at different stages of reproduction.

## 2. Results

### 2.1. Samples

**Figure 1:**
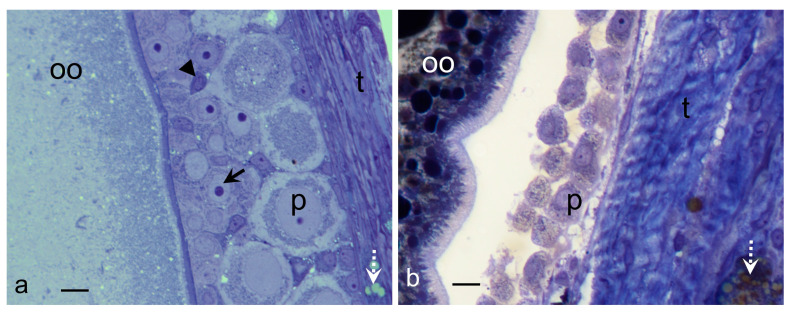
Reproductive stages seen in the ovaries of *S. torquatus*. Toluidine Blue, Bright field. (**a**) Follicle in EV; (**b**) Follicle in LV. Arrow, intermediate cell; arrowhead, small cell; dotted arrow, lipid droplets; oo, ooplasm; p, piriform cell; t, theca. Bars: 10 µm.

Samples of the middle oviduct and liver of the viviparous lizard *Sceloporus torquatus* in two stages of the reproductive cycle were selected: early vitellogenesis (EV) and late vitellogenesis (LV). The reproductive stages were identified according to the morphological, histological, and histochemistry conditions of their gonads [54] (Figure 1). The follicles in EV showed an oocyte with a vacuolated ooplasm and acidophilic staining in the periphery (Masson’s Trichrome). Additionally, it had a thin differentiated zona pellucida, a granulose layer with the three typical cell types: small cells, intermediate cells, and piriform cells, and a thick thecal layer (Figure 1a). The follicles in LV showed an ooplasm full of yolk platelets, a thick and differentiated zona pellucida, a granulose layer on its way to regression to a monolayer, composed solely of piriform cells, and its thecal layer was thicker than those of the follicles in EV (Figure 1b).

### 2.2. Immunofluorescence and Immunoelectron Localization of Splicing Factors in Several Reptile Cell Types

**Figure 2:**
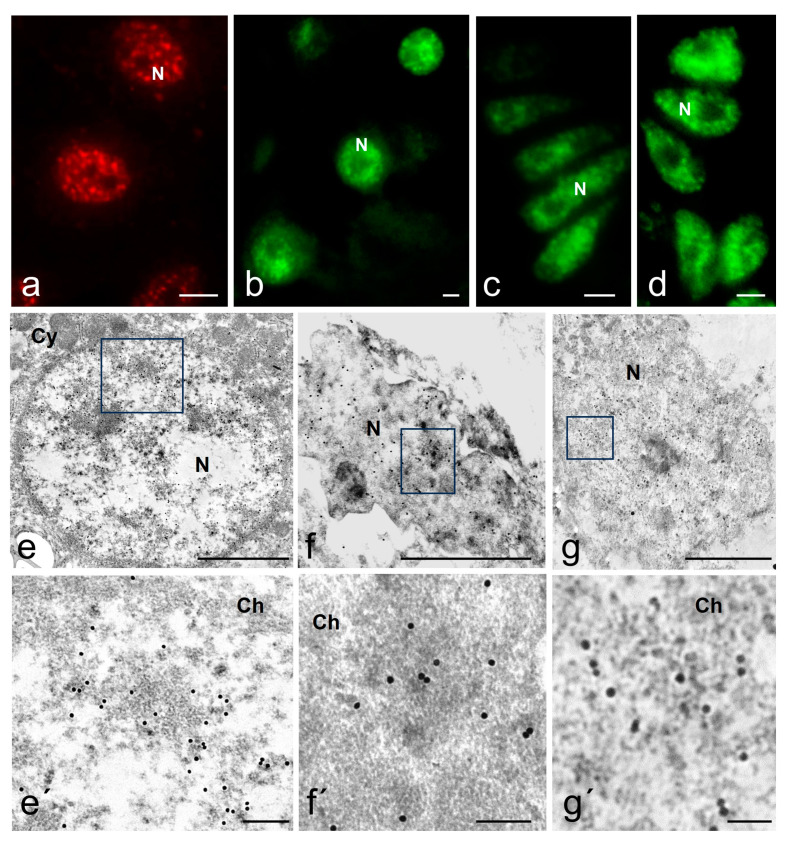
Immunofluorescence and immunoelectron localization of splicing factors in several reptile cell types. (**a**) Control, immunofluorescence of splicing factors in a mammal human lung fibroblast. (**b**–**d**) Fluorescence immunolocalization of the reptile *S. torquatus* cells: (**b**) Hepatocyte; (**c**) Luminal epithelial cells of the oviduct; (**d**) Glandular epithelial cells of the oviduct. Immunoelectron microscopy of splicing factors of reptile cells of *S. torquatus*. (**e**) low and (**e’**) high magnification of a hepatocyte cell nucleus; (**f**) low and (**f’**) high magnification of an oviduct luminal epithelial cell nucleus; (**g**) low and (**g’**) high magnification of an oviduct glandular epithelial cell nucleus. (**a**) Bar is 10 μm; (**b**–**d**) Bar is 2 μm; (**e**–**g**) Bar is 2 μm; (**e’**–**g’**) Bar is 200 nm. Cy, cytoplasm, N, nucleus, Ch, chromatin.

The antibody used in the fluorescence immunolocalization assay recognizes a speckled pattern in mammalian cells (Figure 2a) and also in the reptile cells used in this study (Figure 2b–d). Regardless of reproductive condition and cell type, all cells analyzed in this study presented a positive antibody labeling in the nucleoplasm, which was absent in the nucleolus (Figure 2b–d). The pattern in the nucleoplasm includes the presence of intensely stained speckles and a diffuse pattern of lighter and homogeneous staining as described in mammalian cells. Regardless of cell state, *S. torquatus* oviductal cells (Figure 2c,d) presented an average of 7.6 to 9.3 speckles per cell, with a maximum of 27. Their average cross-sectional area varied between 0.477 and 0.811 μm^2^. Additionally, hepatocytes (Figure 2b) showed an average of 11.2 speckles, with a maximum of 26 per cell. These had an average cross-sectional area of 0.387 μm^2^.

**Figure 3:**
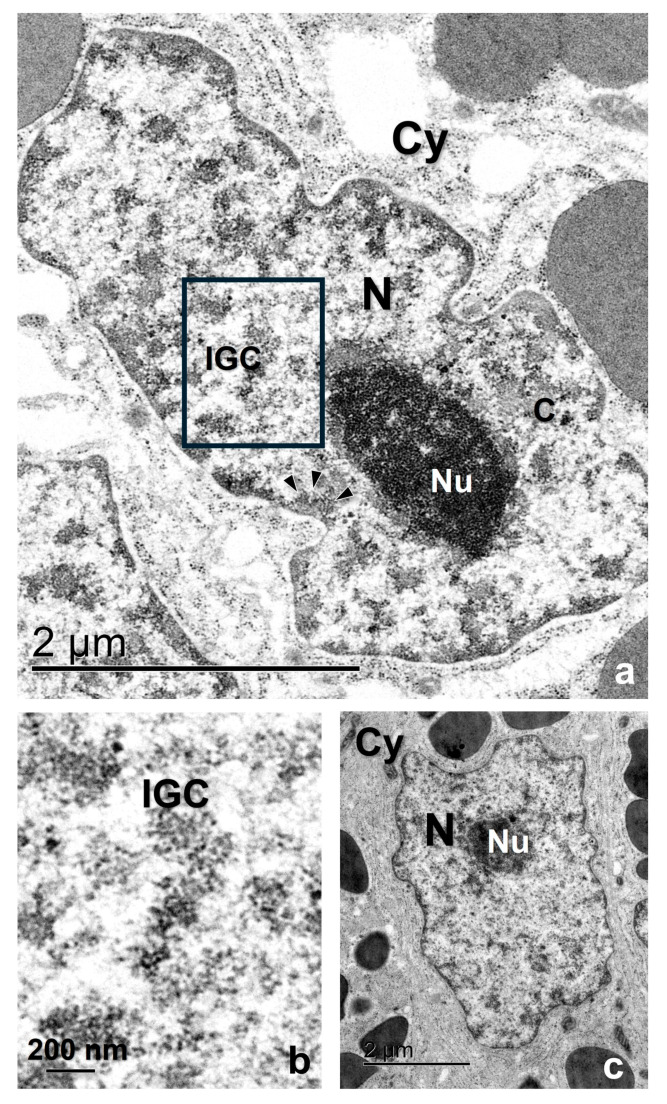
Glandular cells of the oviduct of *S. torquatus.* (**a**) EDTA contrasted cells showing different RNP structures in the nucleoplasm. (**b**) High magnification of the square area. (**c**) Uranyl-Lead contrasted cell showing the normal ultrastructure of the cell. Arrowhead, PF; C, chromatin; Cy, cytoplasm; N, nucleus, Nu, nucleolus, IGC, interchromatin granule cluster.

**Figure 4:**
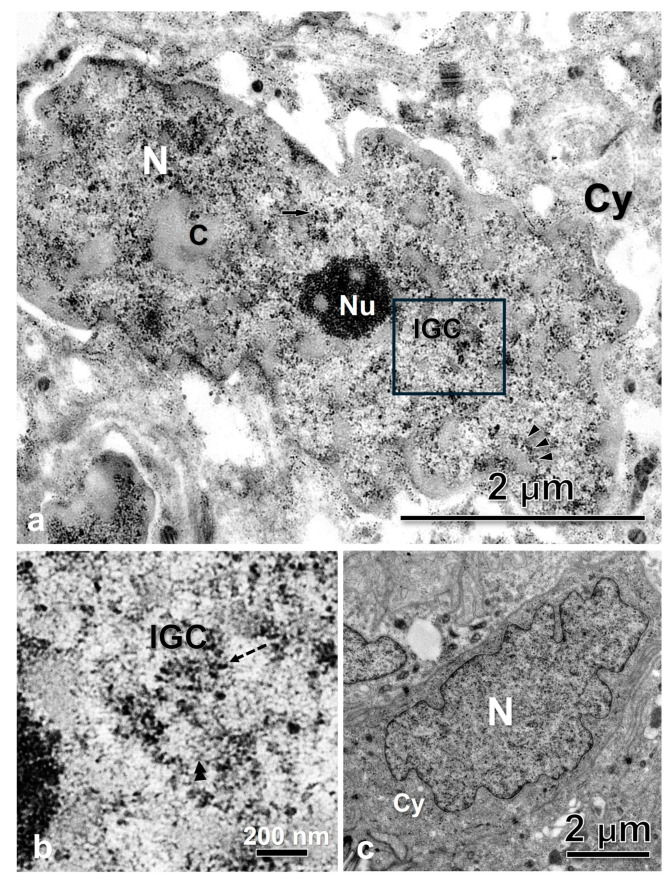
Luminal epithelial cells of the oviduct of *S. torquatus.* (**a**) EDTA contrasted cells showing different RNP structures in the nucleoplasm. (**b**) High magnification of the square area. (**c**) Uranyl-Lead contrasted cell showing the normal ultrastructure of the cell. Arrowheads, PF; arrow, PG; C, chromatin; Cy, cytoplasm; dashed arrow, Interchromatin granule; IGC, interchromatin granules cluster; N, nucleus, Nu, nucleolus.

**Figure 5:**
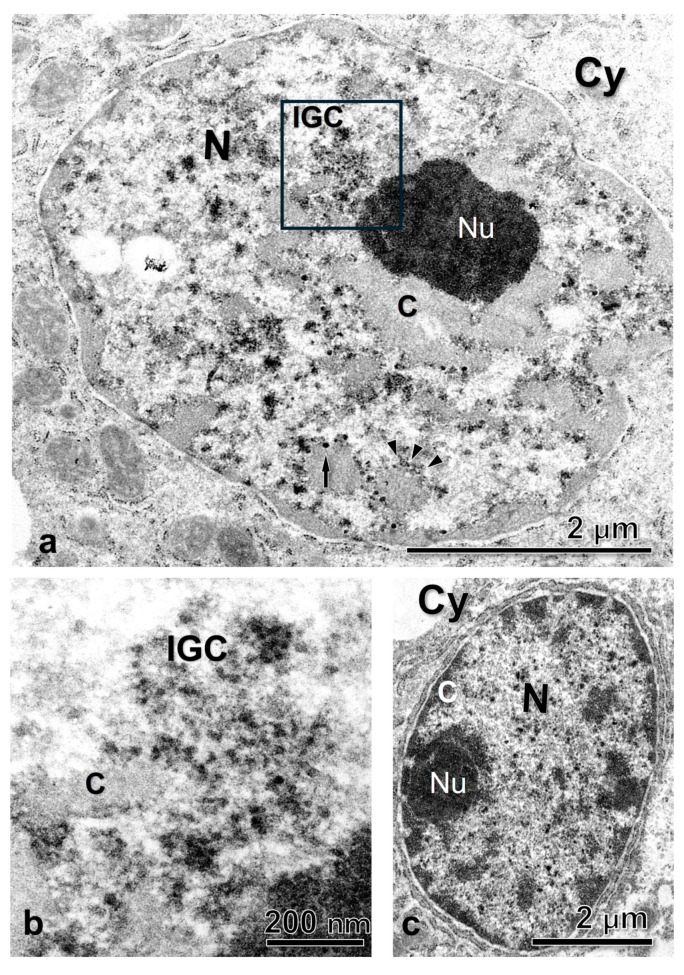
Hepatocytes of *S. torquatus.* (**a**) EDTA contrasted cells showing different RNP structures in the nucleoplasm. (**b**) High magnification of the square area. (**c**) Uranyl-Lead contrasted cell showing the normal ultrastructure of the cell. Arrowhead, PF; arrow, PG; C, chromatin; Cy, cytoplasm; N, nucleus, Nu, nucleolus; IGC, interchromatin granules cluster.

At an ultrastructural level, the Ab recognizes areas in the interchromatin region of the nucleoplasm as well as in the periphery of the condensed chromatin (Figure 2e–g). Using the EDTA contrasting technique developed by Bernhard [55] that allowed us to identify the RNP elements in the nucleoplasm, we found that the interchromatin regions marked by the Ab are of fibrogranular appearance and correspond with the IGCs, and the regions in the chromatin periphery with the PFs (Figure 3, Figure 4 and Figure 5a,b). The Interchromatin Granules showed a mean diameter of 18.66 ± 3.90 nm (*n* = 250). Additionally, we find other RNP particles such as Perichromatin Granules (PGs).

The conservation of the general ultrastructure of each cell type is shown in the uranyl-lead contrasted tissues (Figure 3, Figure 4 and Figure 5c).

### 2.3. Distribution of Speckles in Different Reproductive Stages of the Lizard

**Figure 6:**
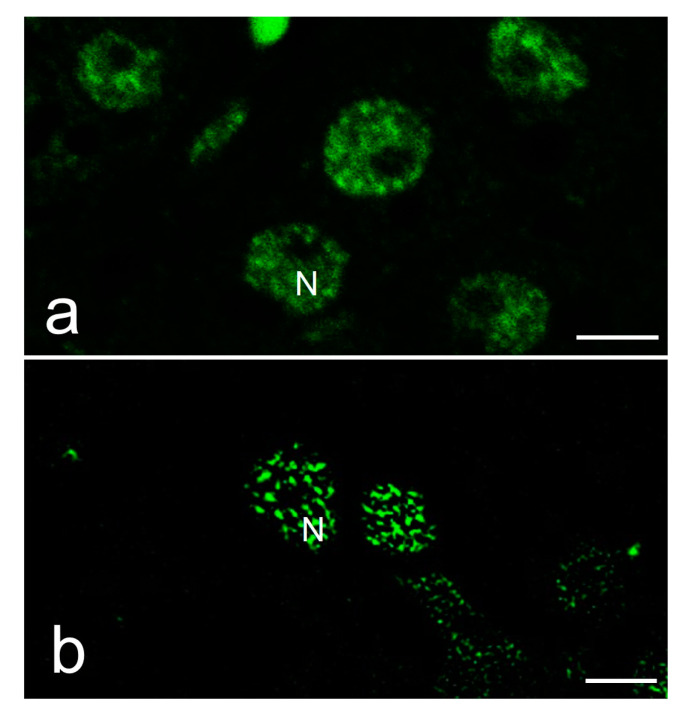
Immunofluorescence of splicing factors in reptile hepatocytes. (**a**) Confocal image. (**b**) MSSR image. N, nucleus, bars: 5 µm.

In order to better analyze the speckled patterns, we enhance the fluorescence labeling by confocal microscopy followed by a procedure of image processing that uses a super-resolution algorithm recently described, known as MSSR [56]. We validate this procedure by showing the comparison of a confocal and an MSSR image (Figure 6). Therefore, we used MSSR images to illustrate further results in this work.

#### 2.3.1. Oviduct

We analyzed the variations in the nuclear speckles in different cell types of the middle oviduct of two females of *S. torquatus*.

**Figure 7:**
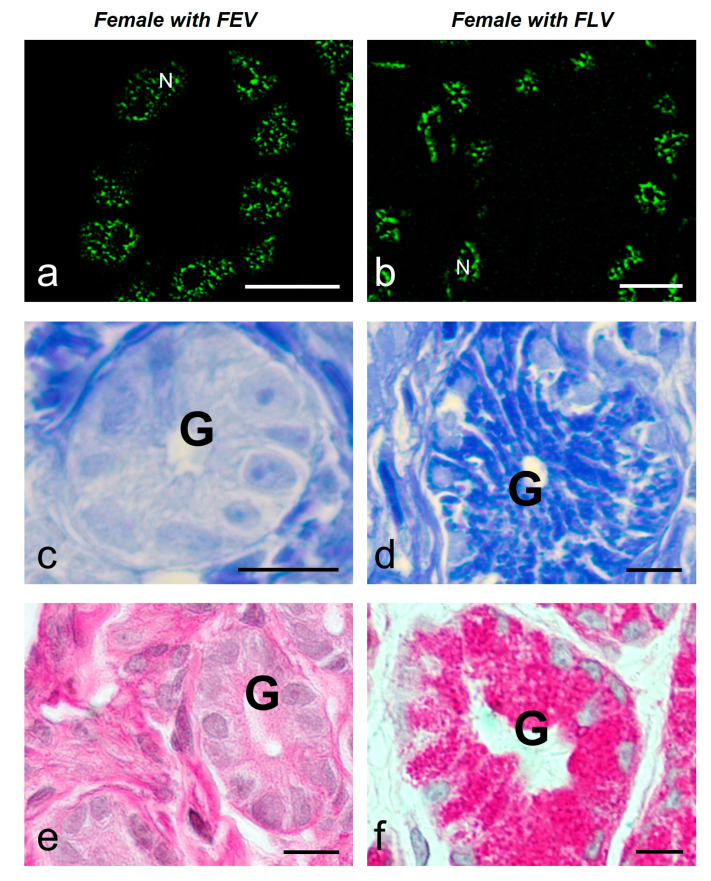
MSSR images of splicing factors in glandular cells of the oviduct of a female with FEV (**a**) and a female with FLV (**b**) of *S. torquatus*. At EV, a pale staining is observed with Coomassie Blue (**c**) and PAS (**e**) histochemistry in the glands (G). At LV, a strong staining is observed with Coomassie Blue (**d**) and PAS (**f**) histochemistry. N, nucleus; bars: 10 μm.

**Figure 8:**
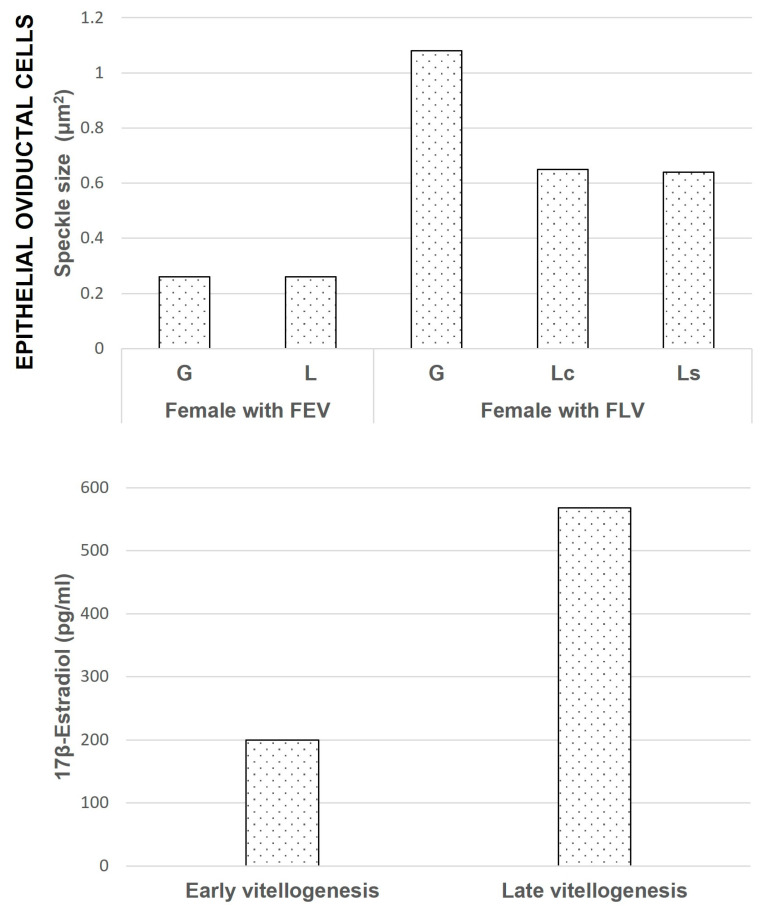
The size of speckles in the oviductal cells in relation to reported [57] hormonal levels during the reproductive cycle of *S. torquatus*. In the glandular and luminal epithelial cells, the size of speckles is much higher in the female with FLV than in the Female with FEV. G: Glandular cell, L: Luminal cell, Lc: Ciliated luminal cell, Ls: Secretory luminal cell.

The glandular epithelial cells (*n* = 80) showed a similar average number of speckles per cell in both animals (8.9 ± 4.6 and 7.0 ± 4.1). The average size of the speckles was smaller and more regular in the female with follicles in EV (FEV) (0.26 ± 0.09 μm^2^) than in the female with follicles in LV (FLV) (1.09 ± 1.01 μm^2^) (with significant difference) (Figure 7a,b) (Figure 8). The glandular cells of the females with FEV presented a negative response to the Coomassie Blue and Periodic Acid Schiff (PAS) reactions (Figure 7c,e), while the same ones in the female with FLV presented a positive reaction to Coomassie Blue staining for proteins in their cytoplasm and a very intense reaction to PAS (Figure 7d,f).

**Figure 9:**
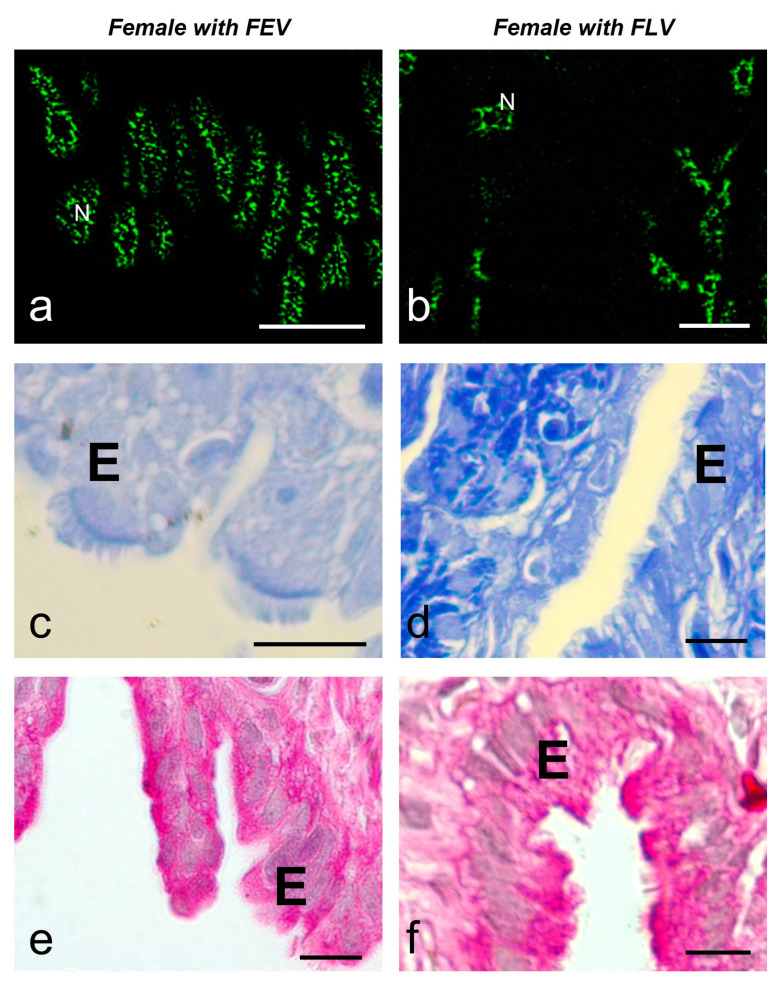
MSSR images of splicing factors in luminal epithelial cells of the oviduct of a female with FEV (**a**) and a female with FLV (**b**) of *S. torquatus*. At EV stage, a pale staining is observed with Coomassie Blue (**c**) and PAS (**e**) histochemistry in the epithelium (E). At LV, a strong staining is observed with Coomassie Blue (**d**) and PAS (**f**) histochemistry. N, nucleus; bars: 10 μm.

The luminal epithelium (*n* = 120) showed different morphology according to the stage of vitellogenesis. In the female with FEV, the epithelium was 10.43 ± 1.13 μm high and showed a predominance of cuboidal–columnar cells with microvilli on their apical face, occasionally bearing cilia. In the female with FLV, the epithelium was higher (19.09 ± 1.94 μm) and showed columnar ciliated cells with elongated nuclei, interspersed with secretory cells with basal nuclei (Figure 9c–f).

The average number of speckles in the ciliated cells of the luminal epithelium was similar in both females (9.9 ± 3.9 and 11.1 ± 5.9), showing significant differences between them and the secretory cells of the female with FLV (6.9 ± 3.0). The average size of speckles was significantly different and smaller in the female with FEV (0.26 ± 0.07 μm^2^) than in the female with FLV (ciliated 0.65 ± 0.32 μm^2^ and secretory 0.84 ± 0.60 μm^2^). (Figure 9a,b) (Figure 8). These cells also differ in their histochemistry; in the female with FEV, the Coomassie Blue staining was negative (Figure 9c) versus a slightly positive staining in the female with FLV (Figure 9d). The PAS reaction was positive in the luminal cells of both females, although it was more intense in the secretory cells (Figure 9e,f).

#### 2.3.2. Liver

**Figure 10:**
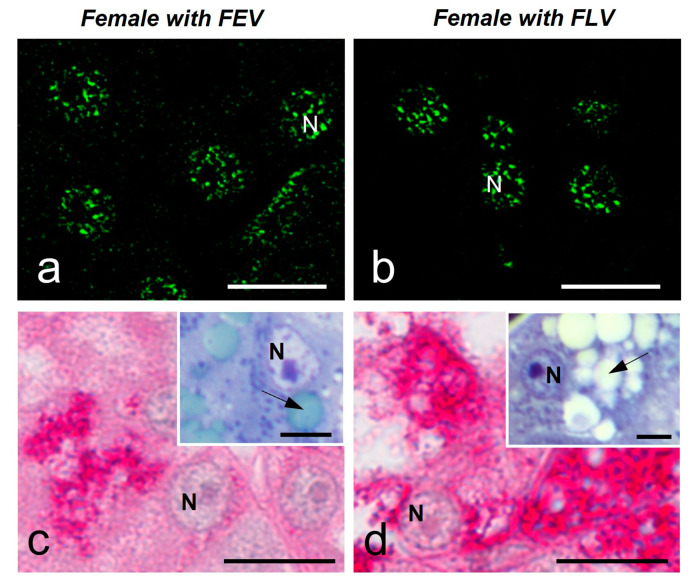
MSSR images of splicing factors in hepatocytes of a female with FEV (**a**) and a female with FLV (**b**) of *S. torquatus*. PAS staining increases from EV (**c**) to LV (**d**). Toluidine Blue staining shows low and large amounts of droplets in the cytoplasm of the hepatocytes of females with FEV and with FLV, respectively (**c**,**d**, insets, arrows). N, nucleus; bars: 10 μm, insets: 5 μm.

In order to evaluate the speckled pattern in another organ involved to some extent in the reproductive functions of reptiles, we study the liver. We analyzed the hepatocytes of both females in the study. In both cases, the speckled pattern was similar (Figure 10a,b) (Table 1). At these stages, hepatocytes show a positive, strong PAS reaction (Figure 10c,d). In addition, the cytoplasm of hepatocytes presents droplets in the cytoplasm as detected by Toluidine Blue staining of semithin sections (Figure 10c,d, insets).

## 3. Discussion

We have shown that speckled patterns of splicing factors are present in reptile cell nuclei and that these patterns show morphological differences depending upon the reproductive stage of lizard *Sceloporus torquatus* females.

### 3.1. Speckled Pattern of Splicing Factors Are Present in Reptile Cell Nuclei

The immunolocalization of the protein splicing factors carried out in the present study used a primary monoclonal antibody that recognizes four of the SR proteins that accumulate in the nuclear speckles, namely SRSF4, SRSF5, SRSF6, and SRSF7 [58] in several species. In addition, it recognizes 20 other nuclear proteins, including U1 70K (a component of U1 snRNP) and the two subunits of U2AF1. All these proteins are involved in pre-mRNA processing, and their presence in nuclear speckles has been previously recognized [35].

The distribution of these proteins in the nucleoplasm and not in the nucleolus, in a pattern of intense speckles immersed in more homogeneous diffuse staining, confirms the existence of the speckled pattern of splicing factors [15,59] in somatic cells of a non-mammalian vertebrate, as already reported [60]. Several cell types display the speckled pattern in the lizard, i.e., hepatocytes, oviduct gland, and luminal epithelial cells. Lizard cells display up to 26 speckles per cell, which is similar to the reported number of 25–50 speckles per cell in mammalian culture cells [1] and in the tissue cells of the rat [4]. However, speckle size in reptilian cells is smaller than that reported for mammals, maybe due to the small size of cells.

To better analyze the speckled pattern at the ultrastructure level, we used the preferential technique for RNP developed by Bernhard [55] that is widely used in the analysis of RNP-containing structures within the nucleus [3,7,15,61,62], and the ultrastructure immunolocalization with the anti-SR proteins Ab. As expected, the EDTA technique revealed the presence of RNP structures in the nucleoplasm that we identified as IGCs and PFs [3,25,63].

The IGCs appear as fibrogranular networks in the interchromatin space and are, in some cases, in close association with clumps of chromatin, as observed in Figure 5. The association between the nuclear speckles components and the chromatin has been reported as a stable and strong biochemical association [64].

Although the conservation of the tissues used for the ultrastructure immunolocalization was not optimal given the fixation procedure used (paraformaldehyde 4% instead of the paraformaldehyde-glutaraldehyde mixture, in order to preserve the antigen epitope), it was possible to discern that both IGCs and PFs display the gold particles indicating the presence of the SR proteins in their composition.

Moreover, some other RNP nuclear particles, such as Perichromatin Granules [65], were observed repeatedly in the nucleoplasm of the different cell types. Further work is required to unravel the significance of their presence, morphology, and variations in number, among other aspects, in the reptilian cells.

### 3.2. Morphology of Speckled Pattern in Reptile Oviductal Cells Changes upon the Reproductive Stage

In order to evaluate whether cells exposed to hormones during the reproductive cycle in reptiles behave as those in mammals, we decided to work with the viviparous lizard *S. torquatus*, which presents a middle oviduct also named glandular uterus with gland and luminal epithelium [66]. Our results show that the speckled pattern of splicing factors found in oviductal reptilian tissues varies according to the reproductive condition of the specimen in both gland and luminal epithelia.

In the oviductal epithelium, the speckled pattern displays smaller speckles in the female with FEV than in the female with FLV. There is a different physiological condition in every stage. In fact, the morphological, histological, and histochemical conditions of the oviductal cells in reptiles change along the reproductive cycle, and those changes are mediated by a rise of estradiol (E2) in plasma together with the expression of E2 receptors in the oviductal tissues, as previously reported [67,68]. These changes allow the middle or glandular oviduct to fulfill its function in embryo incubation and deposit of the shell membranes [66,69,70]. Among the mentioned modifications are an increase in epithelial width, differentiation of the cell populations of the luminal epithelial, and an increase in the number and activity of the glands [66]. We found that the oviduct of the female with FEV presents evidence of early stages of preparation for reproduction, as suggested by its lower epithelium, composed of undifferentiated cells and the absence of material accumulated in its glands. The level of plasma E2 for the species in this reproductive stage is increasing, but it has not yet reached a peak [57]. The glandular and luminal cells of the oviduct of this animal showed smaller and more compact speckles.

On the other hand, in the female with FLV, the width of the luminal epithelium of the oviduct is higher, there is a differentiation of its cell populations into ciliated and secretory ones, as well as the characteristics of a more considerable number and size of the glands, and evidence of accumulation of proteins and PAS-positive material that is only shown in the final phases of vitellogenesis [71], along with a peak in the level of the circulating hormone [57]. The oviductal cells show larger and irregular speckles. The increase in size, together with the irregular and amorphous morphology of the speckles in the cells of the active stage of reproduction, may be due to an increase in the fluidity of the speckle’s components. The relation between the morphology and the fluidity has been previously reported [43]. It would require further work and advanced techniques to analyze if the transit of transcripts on their way to the cytosol mentioned by some authors [72,73] may also be influencing the size of the speckle in a cell that is actively involved in the synthesis of protein products for reproduction [71].

Our results agree with those previously described for mammals [4], where changes were detected in the speckled pattern in the luminal and glandular epithelia of the rat uterus, depending upon the estrus cycle, as well in experiments of castration and further injection of E2 to control the hormone, leading to the conclusion that the action of steroid hormones may mediate the changes in the speckled pattern. The same situation may occur in reptiles, given that their reproductive cycle is mediated by steroid hormones in a way similar to mammals [74]. In *S. torquatus*, the E2 concentration during the reproductive cycle has been reported [57]. The concentration is lower in the early than during the late vitellogenic stages. We propose, accordingly, that in reptiles, the changes in the morphology of the speckled pattern of splicing factors in oviductal cells depend on the reproductive condition because of steroid hormones, as in mammals.

### 3.3. Morphology of Speckled Pattern in Reptile Hepatocytes Does Not Change upon the Reproductive Stage

The liver histological features of the specimens in our study reflect its role in the metabolism of nutrition [75,76] and the event of vitellogenesis in females [77,78]. The liver of female amphibians, reptiles, and birds has a recognized role in vitellogenesis; it is the organ responsible for the synthesis, processing, glycosylation, and release of vitellogenin, which is internalized to the oocyte [79,80,81]. The morphological variations of the liver imply changes in the size of the hepatocytes [82], the presence of glycogen [76], and the accumulation of lipids [77,83], among others.

The lipid accumulation visualized by the presence of cytoplasmic droplets in females was higher in the female with FLV, in agreement with the increase in lipid content in the liver reported in reptiles during the vitellogenesis, which is mediated by the rise of plasma E2 [84].

The lack of differences in the speckled pattern in the hepatocytes of females in different stages of vitellogenesis may be due to a restricted effect of the E2 in the liver, which may be focused on the induction of transcription of the vitellogenin gene and the post-translational modifications of the proteins [85,86].

### 3.4. Final Considerations

Our results are consistent with the notion that the morphology of the speckled pattern is modified in culture and tissue cells submitted to different conditions that modify the transcriptional and splicing activities, including the hormone effect during the estrus cycle in mammals. We now extend this model to other vertebrates as a viviparous reptile.

## 4. Materials and Methods

Two specimens of *Sceloporus torquatus* were collected in the REPSA in Mexico City during the fall of 2022. Immediately after the collection, the specimens were sacrificed through an overdose of sodium pentobarbital (Dolethal) with previous sedation with Midazolam and Ketamine. Once the absence of heart rhythm was verified using a Doppler ultrasound, the animals were dissected, and ovarian, liver, and oviduct fragments were fixed according to the needs of each processing, which will be described below. The bodies and other tissues of the animals, which were not used for this study, were donated to the Wildlife Group of the REPSA’s Executive Secretariat (SEREPSA). To carry out the collections, euthanasia, and dissection procedures, we had the collection permit from SEMARNAT (Office No SGPA/DGVS/02556/22), entry and collection permit granted by the SEREPSA (Office: REPSA/92/2022, Project 583) and the opinion approving the project by the Ethics and Scientific Responsibility Committee of the Faculty of Sciences of the UNAM (Office: CEARC/Bioética/12062022).

### 4.1. Characterization of Tissues

We implemented the General Histological Technique (GHT) to analyze the tissue’s general structure. The tissue samples were fixed by immersion in 4% paraformaldehyde for 2 h and then dehydrated using ethanol of increasing concentrations (30%, 50%, 60%, 70%, 80%, 90%, and 100%) to continue clearing in xylol and the inclusion in paraffin. We obtained sections between 3 and 5 μm thick in previously gelatinized slides with a manually rotating microtome. Finally, the slides were stained with the Periodic Acid Shift Reaction (PAS), Masson´s Trichrome, and Coomassie Blue for protein staining [87].

Also, we performed the classical processing technique for electron microscopy: duplicated fragments of 1 mm^3^ of the tissues were fixed with Karnovsky’s mixture (2.5% glutaraldehyde-4% paraformaldehyde) for one hour, followed by a post-fixation step with osmium tetraoxide (OsO_4_) on one set of the tissue samples. Then, gradual dehydration was carried out using increasing concentrations of ethanol, followed by propylene oxide as an intermediate agent and slow pre-inclusion with mixtures of propylene oxide and epoxy resin. Finally, it was included in epoxy resin in a silicone mold and allowed to polymerize in the oven for 24 h at 60 °C.

Using a Leica Ultracut RT ultramicrotome, 300 to 500 nm semi-thin sections were obtained and stained with Toluidine Blue to select the areas of interest. The stained sections were observed and photographed under a Nikon E800 microscope (New York, NY, USA) using the NIS Elements D software v5.30.01.

Ultra-thin sections of an approximate thickness of 60 nm on formvar-coated copper grids were obtained using the blocks with the tissues of the post-fixed set. They were contrasted with the general uranyl acetate—lead citrate technique (20′-10′, respectively). All the grids were observed in the JEOL (Tokyo, Japan) JEM-1010 (80 kV) and recorded with a CCD model Gatan (Pleasanton, CA, USA) Orius SC600 cameras.

### 4.2. Fluorescent Immunolocalization

Sections obtained with the previously described GHT were used to carry out the fluorescent immunolocalization of the SR proteins. The slides were rehydrated with ethanol in decreasing concentrations (100%, 96%, 90%, 70%, and 50%) and finally in distilled water. Then the Antigen retrieval protocol with citrate buffer was implemented according to the AbCam protocol ([https://www.abcam.com/protocols/ihc-antigen-retrieval-protocol], accessed on 22 April 2021). After that, the tissues were blocked with BSA (5%) for 2 h. Then, incubation was carried out with the mouse monoclonal antibody (Ab) anti-SR proteins (Non-snRNP Splicing Factor) (USBiological, Cat No.: S6570, Salem, MA, USA), with a 1:100 dilution, overnight, in a humid chamber at 4 °C. After incubation, the slides were washed with TBST buffer to remove excess Ab and incubated with the secondary Ab, a polyclonal rabbit anti-mouse immunoglobulin coupled to Texas Red or FITC (Dako, R0270 or F0232, Nowy Sącz, Poland). A 1:20 dilution was used, and the incubation was made for one hour in a dark, humid chamber at room temperature. The slides were finally washed with TBST buffer. The slides were mounted with Vectashield fluorescence mounting medium (H-1000) and observed and photographed in a Nikon E800 microscope using the NIS Elements software v5.30.01 and a confocal microscope, Olympus FV1000 using the software Olympus Fluoview v4.0.

### 4.3. Ultrastructural Cytochemical Analysis

In order to evaluate the RNP elements, a cytochemical ultrastructural protocol was conducted in ultra-thin sections of an approximate thickness of 60 nm on formvar-coated copper grids obtained from the blocks with the non-post-fixated tissues. The regression technique for ribonucleoproteins with uranyl acetate—ethylenediaminetetraacetic acid (EDTA)—lead citrate (3′-18′-2′, respectively) was performed. All the grids were observed in the JEOL JEM-1010 (80 kV) and recorded with a CCD model Gatan Orius SC600 camera.

### 4.4. Ultrastructural Immunolocalization

In order to confirm the presence of SR proteins in the IGC at an ultrastructural level, we performed an ultrastructural immunolocalization. Tissue fragments were fixed by immersion in 4% paraformaldehyde for one hour, rinsed in PBS, and then dehydrated using methanol of increasing concentrations [30%, 50% at 4 °C and 70%, 90% at −20 °C). The slow pre-inclusion process was carried out with mixtures of methanol 90% and Lowicryl K4M acrylic resin, ending with three steps of Lowicryl at −20 °C. Finally, it was included in closed gelatin capsules and allowed to polymerize in a UV chamber for 24 h at −20 °C.

Using a Leica (Wetzlar, Germania) Ultracut RT ultramicrotome, sections with an approximate thickness of 60 nm on nickel grids were obtained. The incubation was carried out with the same anti-SR proteins Ab used in the fluorescence immunolocalization, with a 1:10 dilution, overnight, in a humid chamber at 4 °C. After incubation, the grids were washed with TBST buffer to remove excess Ab and incubated with the secondary Ab, a polyclonal goat anti-mouse immunoglobulin coupled to colloidal gold of 20 nm (Zymed). A 1:20 dilution was used, and the incubation was made overnight in a humid chamber at 4 °C. The grids were finally washed with TBST buffer and double-distilled water and contrasted with uranyl acetate for 10 min. They were observed in the JEOL JEM-1010 (80 kV) and recorded with a CCD model Gatan Orius SC600 camera.

### 4.5. Image Analysis

The images obtained by optical microscopy (bright field and fluorescence) were analyzed with the Fiji software version 1.53t.

The fluorescence images were processed with the recently published Mean-Shift Super Resolution algorithm (MSSR) using the MSSR 2.0 plugin at Fiji. MSSR is a deconvolution algorithm that allows us to increase the resolution of the fluorescence signal and denoise the image [56]. The parameters employed were MSSR of Order 1, with an Amplification of 2 and a full width at half maximum (FWHM) calculated for each image with the Image Decorrelation Analysis plugin (also accessible in Fiji).

After that, an automatic segmentation threshold was applied to each cell, and the Particle analysis tool was used to measure the area of the elements.

The statistical analysis was carried out using the software Statistica v.10.

The sample means of the parameters were compared using a Simple Parametric Variance Analysis (ANOVA) for those that meet the premises of normality and homogeneity of variance. A posteriori comparison test was carried out employing a Tukey test for the comparisons where the Ho was rejected. Data that did not meet the premises for parametric tests were compared using the non-parametric Kruskal-Wallis ANOVA.

## Figures and Tables

**Table 1:** Number and size of the speckles in the hepatocytes of *S. torquatus*. Values are presented as Mean ± Standard Deviation (*n* = 100).

	Early Vitellogenesis	Late Vitellogenesis
Number of speckles	12.3 ± 3.9	10.4 ± 5.3
Speckle size (µm^2^)	0.24 ± 0.08	0.29 ± 0.18

## Data Availability

The original contributions presented in the study are included in the article; further inquiries can be directed to the corresponding author.
